# Repeated Episodes of Heroin Cause Enduring Alterations of Circadian Activity in Protracted Abstinence

**DOI:** 10.3390/brainsci2030421

**Published:** 2012-09-20

**Authors:** Luis Stinus, Martine Cador, Stephanie Caille

**Affiliations:** 1 University of Bordeaux, INCIA, F-33076 Bordeaux, France; Email: luis.stinus.saez@gmail.com (L.S.); martine.cador@u-bordeaux2.fr (M.C.); 2 National Center of Scientific Research (CNRS), UMR 5287, INCIA, F-33076 Bordeaux, France

**Keywords:** opiate, dependence, protracted abstinence, circadian activity, rats

## Abstract

Opiate withdrawal is followed by a protracted abstinence syndrome consisting of craving and physiological changes. However, few studies have been dedicated to both the characterization and understanding of these long-term alterations in post-dependent subjects. The aim of the present study was to develop an opiate dependence model, which induces long-lasting behavioral changes in abstinent rats. Here, we first compared the effects of several protocols for the induction of opiate dependence (morphine pellets, repeated morphine or heroin injections) on the subsequent response to heroin challenges (0.25 mg/kg) at different time points during abstinence (3, 6, 9 and 18 weeks). In a second set of experiments, rats were exposed to increasing doses of heroin and subsequently monitored for general circadian activity up to 20 weeks of abstinence. Results show that heroin injections rather than the other methods of opiate administration have long-term consequences on rats’ sensitivity to heroin with its psychostimulant effects persisting up to 18 weeks of abstinence. Moreover, intermittent episodes of heroin dependence rather than a single exposure produce enduring alteration of the basal circadian activity both upon heroin cessation and protracted abstinence. Altogether, these findings suggest that the induction of heroin dependence through intermittent increasing heroin injections is the optimal method to model long-term behavioral alterations during protracted abstinence in rats. This animal model would be useful in further characterizing long-lasting changes in post-dependent subjects to help understand the prolonged vulnerability to relapse.

## 1. Introduction

Drug dependence—or addiction—is often described as a chronic mental disorder characterized by compulsive drug intake and loss of control over intake despite adverse consequences [1,2,3,4]. Moreover, the fact that vulnerability to relapse in addicts can persist after many years of abstinence implies that addiction is caused by long-lasting changes in brain function as a result of pharmacological insult (repeated remission and relapse episodes), genetic predisposition, and environmental associations made with drug use (learning). Thus, a primary problem for the treatment of drug abuse remains to be the understanding of the brain neuroadaptations following repeated drug use that might be responsible for the persistent vulnerability to drug craving and seeking after abstinence [5,6].

In humans, the average period of time to relapse to opiate use is 25 days, while the rate of relapse is 71% within the first 6 weeks and 95% within 3 months [7]. The high rate of relapse to opiate abuse in this time frame is closely related to prolonged vulnerability to craving, sleep disturbances, dysphoria and anhedonia, a cluster of symptoms called the protracted withdrawal syndrome [8,9]. General circadian activity, a correlate of arousal and sleep periods in humans, has been studied in animal models of opiate dependence. It has been shown that similar to humans [8,10], abrupt drug cessation can alter the circadian biphasic activity for several days during abstinence in animal models of opiate dependence [11,12,13].

A large body of evidence suggests that the consequences of opiate withdrawal and conditioned withdrawal support both maintenance of drug taking and relapse to opiate seeking [14,15,16,17,18]. On one hand, chronically morphine-pretreated abstinent rats express stronger and longer lasting preferences for morphine-associated environments than placebo-pretreated rats (at least 5 weeks, [19]), develop a progressive incubation of drug seeking behavior over a two-month withdrawal period [20], and a long-lasting hypersensitivity to the psychostimulant effect of an opiate injection [21]. On the other hand, while it has been very difficult to demonstrate the important role of drug withdrawal in the maintenance of, and relapse to drug use [17,22], recent work successfully reported that morphine-withdrawal conditioned cues significantly reinstate drug seeking [23,24]. Likewise, there is a persistence of withdrawal-induced negative motivational affects such as anxiety [25] and aversion up to 16 weeks in abstinent rats [26]. Altogether, these findings highlight how long lasting the vulnerability to opiates is in abstinent rats.

It is common for opiate addicts to experience multiple episodes of compulsive drug taking interspersed by periods of attempted abstinence. Similar to humans, opiate dependence in rodents is a progressive phenomenon that may begin with a single dosing [27], but its severity augments with the increasing number of exposures to the drug [27,28]. For instance, the role of a repeated withdrawal syndrome has been shown to be critical in the escalation of volitional drug intake, including nicotine intake [29]. In addition, morphine dependence provided by a constant infusion or implanted pellets does not activate the hypothalamic-pituitary-adrenal (HPA) axis [30,31], while repeated exposures with escalating doses of opiate alter the stress system responses, a key element in opiate addiction [32,33]. Thus, these data support the hypothesis that repeated cycles of addiction contribute to the development of neuroadaptations involved in the vulnerability to drug addiction.

Thus, our hypothesis was that a robust and repeated exposure to opiates would have more pronounced behavioral alterations during long-term abstinence. The aim of the present study was to show that the diverse drug regimens to induce opiate dependence (morphine pellets, morphine injections or heroin injections) might produce differential locomotor activity responses to a heroin challenge during abstinence. Second, we examined the effect of multiple cycles of heroin dependence on the alterations in circadian locomotor activity during both spontaneous withdrawal and protracted abstinence.

## 2. Results

### 2.1. Experiment 1: Long Lasting Sensitivity to Heroin Challenge Depends on Previous History of Opiate Dependence (Figure 1)

The rats in the group that received placebo pellets and in the group that received saline injections did not differ on any behavioral measure. Thus, their data were pooled and presented as the control group.

**Figure 1:**
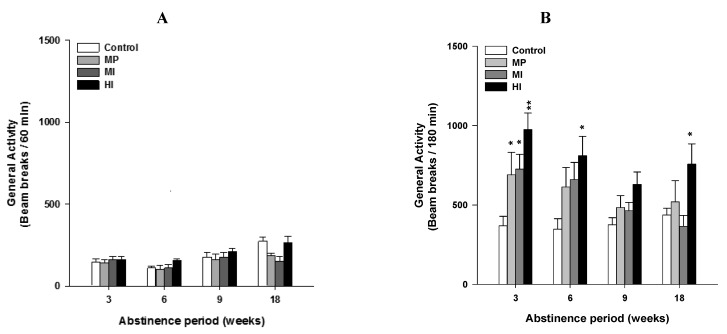
Total locomotor activity in response to (**A**) saline and (**B**) heroin peripheral challenges in opiate abstinent rats. Challenges were performed at 3, 6, 9 and 18 weeks of abstinence in animals abstinent from morphine pellets (MP), morphine injections (MI), heroin injections (HI), and in control animals (Control). ANOVA with repeated measures show no significant statistical interaction. Bar graph (**A**) represents mean ± SEM total number of beam breaks over 60 min in response to a saline challenge (1mL/kg s.c.). Bar graph (**B**) represents mean ± SEM total number of beam breaks over 180 min in response to a heroin challenge (0.25 mg/kg s.c.). Abstinent rats with previous history of heroin injections show longer lasting sensitivity to heroin challenges (Fisher’s PLSD *post-hoc vs.* control group, * *p* < 0.05, ** *p* < 0.01).

First, results show that the locomotor activity elicited by each saline injection does not depend on previous drug treatment (dependence, *p* = 0.12 n.s; dependence × time interaction, *p* = 0.07 n.s.) (see Figure 1A). On the contrary, previous opiate history significantly alters the sensitivity to acute challenges with heroin 0.25 mg/kg s.c. (dependence *F*(3,27) = 4.12, *p* < 0.01; dependence × time interaction, *F*(9,81) = 2.85, *p* < 0.006) (see Figure 1B). *Post-hoc* analysis shows significant difference between IH and other groups (*vs.* control *p* < 0.01; *vs.* MP *p* < 0.07; *vs.* IM *p* < 0.05).

After 3 weeks of abstinence, all the groups pretreated with opiates are hyperactive following the heroin challenge when compared to the control group (MP *p* < 0.05; MI *p* < 0.05 and HI *p* < 0.001).

At 6 weeks, rats previously implanted with morphine pellets (MP) or injected with morphine (MI) are not different from control rats anymore. HI still shows a greater heroin-induced general activity than control rats (HI *p* < 0.05).

At 9 weeks, the groups are not statistically different. Finally, after 18 weeks of abstinence, the HI group still expresses hyperactivity when challenged with heroin (*post-hoc vs.* control, *p* < 0.05). The general activity of HI is also statistically different from the MI (*p* < 0.05), but not different from MP due to a large individual variability within this group.

### 2.2. Experiment 2: Repeated Episodes of Heroin Dependence: Consequences on the Circadian Activity upon Abrupt Cessation (Figure 2)

**Figure 2:**
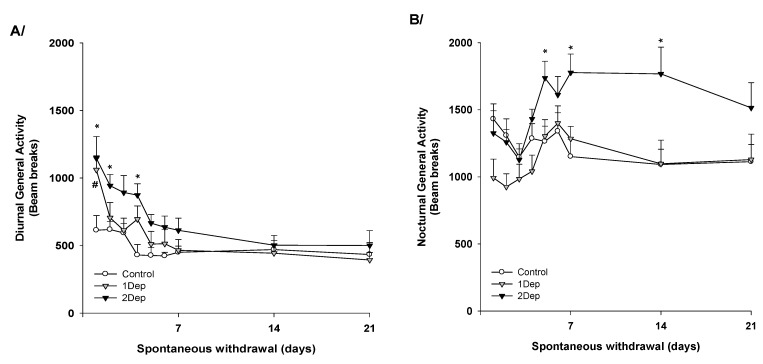
Circadian locomotor activity during spontaneous heroin withdrawal in rats: (**A**) during daytime and (**B**) during nighttime. Activity measures were performed from the first day of treatment cessation to the 21st day of abstinence in animals with a history of single heroin dependence (1Dep), 2 heroin dependence episodes (2Dep) and in control animals (Control). Points represent mean ± SEM. (**A**) Total number of beam breaks during the diurnal part of the circadian cycle. (**B**) Total number of beam breaks during the nocturnal part of the circadian cycle. Spontaneous withdrawal in 1Dep rats disrupts both diurnal and nocturnal activities for couple of days (Fisher’s PLSD *post-hoc vs.* control group, ^# ^*p* < 0.05). Abstinent rats with 2 episodes of dependence show longer lasting sensitivity disruption of both activities when compared to control animals (Fisher’s PLSD *post-hoc*, * *p* < 0.05).

ANOVA shows a significant dependence × withdrawal time-point × circadian activity interaction (*F*(16,120) = 2.57, *p* < 0.01). Specific analysis indicates that during the spontaneous withdrawal period, control rats express the classical biphasic circadian activity (day activity < night activity, *p* < 0.05) while heroin withdrawal disrupts this biphasic activity for a couple of days in both 1Dep and 2Dep groups. In 1Dep rats, diurnal and nocturnal activities are not significantly different from each other on day 1; then from day 2 onwards, daytime activity is lower than nighttime activity (*p* < 0.05). In 2Dep rats, the biphasic activity is disrupted on days 1 and 2 (*p* < 0.05); then from day 3 onwards, daytime activity is lower than nighttime (*p* < 0.05).

Diurnal activity (see Figure 2A). ANOVA over the total time-course indicates a significant heroin effect (*F*(2,15)= 3.44, *p* < 0.05) as well as a significant heroin × time interaction (*F*(16,120) = 1.98, *p* < 0.02). On the first day upon chronic treatment cessation, control rats show an average diurnal activity of 614 ± 108 beam breaks, while both heroin groups show hyperactivity (1Dep 1061 ± 104 counts; 2Dep 1147 ± 160 counts) (control *vs.* 1Dep, *p* < 0.05; *vs.* 2Dep, *p* < 0.05). On day 2 onwards, 1Dep rats return to the same level of diurnal activity as that of control rats (*post-hoc* n.s.). On the other hand, 2Dep rats show abnormally high levels of diurnal activity on days 2 and 4 (*post-hoc p* < 0.05), and eventually, stabilize to the control level on day 5 onwards (*post-hoc* n.s.). 

Nocturnal activity (see Figure 2B). Overall ANOVA over the total time-course indicates a significant heroin effect (*F*(2,15) = 4.07, *p* < 0.05) as well as a significant heroin × time interaction (*F*(16,120) = 2.81, *p* < 0.001). When beginning the spontaneous withdrawal, the 1Dep group expresses a nocturnal level of activity similar to the controls (*post-hoc* n.s). 2Dep group show locomotor activity comparable to the controls’ activity at first (night #1 to night #4, *post-hoc* n.s). Then, 2Dep group shows a drastic long-lasting increase in general nocturnal activity compared to the control group (*post-hoc p* < 0.05 on days 5, 7; and *p* = 0.06 on day 14).

### 2.3. Experiment 3: Effect of Intermittent Exposures to Heroin: A Time-Course Analysis of the Circadian Activity throughout Extended Abstinence (Figure 3)

During the break between the 2 episodes of heroin exposure, rats were not handled except for bedding changes in order to let the spontaneous withdrawal happen without interruptions. When rats were in protracted abstinence (at least 4 weeks post-heroin), experiments and weight monitoring started. The data were the following: Control 578 ± 18 g; 1Dep 559 ± 17 g; 2Dep 508 ± 18 g. Statistical comparison shows a significant difference between the groups (*F*(2,19) = 3,95, *p* < 0.05). 2Dep rats are smaller than the control (Fisher’s PLSD *p* < 0.02) and the 1Dep rats (Fisher’s PLSD *p* < 0.05).

ANOVA shows that for all rats, the circadian activity is biphasic with higher spontaneous activity at night than during daytime (main effect cycle, *F*(1,19) = 417.9, *p* < 0.001). In addition, there is a significant dependence × withdrawal time-point × cycle (*F*(6,57) = 2.91, *p* < 0.01) interaction. Further statistical analyses were done separately on diurnal and nocturnal activities. During daytime, the spontaneous activity levels are dependent on animal pharmacological history (interaction dependence × withdrawal time-point, *F*(6,57) = 2.94, *p* < 0.01; main effect dependence *F*(2,19) = 5.38, *p* < 0.01). Analysis shows that 2Dep group is hyperactive compared to other groups overall (*vs.* control *p* < 0.01; *vs.* 1Dep *p* < 0.01). When comparisons are done for each time point, *post-hoc* analyses indicate that control and 1Dep animals have comparable levels of activity, while 2Dep animals develop higher levels of activity at 16 and 20 weeks of abstinence (*post-hoc vs.* control, *p* < 0.05). At nighttime, there is no significant dependence × withdrawal time-point interaction (*p* value n.s.). However, there is a main effect of dependence (*F*(2,19) = 5.38, *p* < 0.01), 2Dep animals are hyperactive compared to control and 1Dep abstinent rats (*post-hoc* *vs.* control *p* < 0.05; *vs.* 1Dep *p* < 0.001).

**Figure 3:**
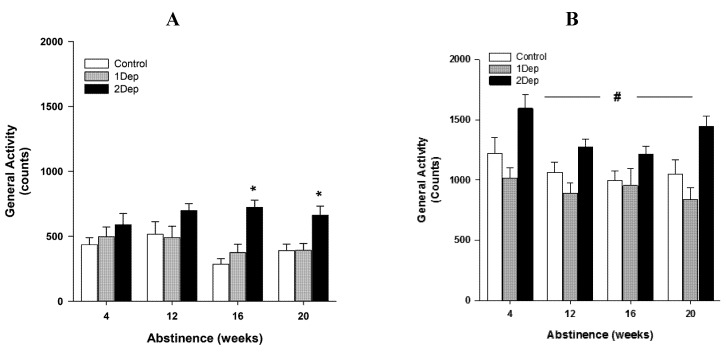
Spontaneous circadian activity at several time-points of heroin protracted abstinence. (**A**) Daytime activity and (**B**) nighttime activity at 4, 12, 16 and 20 weeks in animals with a history of single heroin dependence (1Dep), 2 heroin dependence episodes (2Dep), and in control animals (Control). Points represent mean ± SEM (*n* = 5–7/group). (**A**) Total number of beam breaks during the diurnal part of the circadian cycle. (**B**) Total number of beam breaks during the nocturnal part of the circadian cycle. 2Dep animals show persistent diurnal hyperactivity (Fisher’s PLSD 2Dep *vs.* control group, * *p* < 0.05). Moreover, 2Dep animals show a global increase of nocturnal activity (main effect Dependence, ^# ^*p* < 0.001).

## 3. Discussion

A critical problem in drug addiction is that a majority of addicts return to compulsive drug taking long after acute withdrawal and abstinence. This protracted abstinence state is associated with persistent behavioral and neurobiological abnormalities responsible for drug seeking reinstatement. In the present study, we first show that intermittent episodes of opiate injections rather than constant exposure with morphine pellets lead to longer lasting hyperactivity to heroin challenges (up to 18 weeks). Moreover, the number of dependence episodes is an important factor for the behavioral adaptations. Indeed, post-dependent rats with two episodes of heroin dependence have a disruption of the circadian activity for the first 7 days of spontaneous withdrawal and show abnormal circadian activity patterns up to 18 weeks of abstinence. 

Disruption of the circadian activity is a hallmark of the early opiate withdrawal syndrome in opiate-dependent rats [11,12]. Here, we show that a similar pattern of disrupted circadian activity developed regardless of the opiate (morphine or heroin) and the route of administration (pellet *vs.* injections). It could be suggested that novelty-induced stress by the transfer of the rats into the activity cages may contribute to the cycle disruption. This is unlikely since the present study confirmed our previous work in which rats were habituated to the behavioral paradigm before the induction of opiate dependence [11,12].

Heroin is transformed into morphine once it crosses the blood-brain barrier and therefore produces the same central effects. However, it has been reported that heroin has a more rapid onset of action and is more potent than morphine in producing reinforcing effects in animals [34,35,36]. In the model of intracranial self-stimulation (ICSS), a predictive model for drug abuse liability in rats, it was demonstrated that heroin is 40 times more potent than morphine in lowering the reward threshold [35]. After a rapid deacetylation, heroin is also metabolized into 6-acetylmorphine (6-MAM), a metabolite equipotent to heroin in the ICSS model [35]. In line with these findings, our results indicated that, maybe due to its metabolization into 6-MAM as well as into morphine, heroin rather than morphine induces more persistent changes to subsequent opiate challenges during protracted abstinence. Indeed, hyperactivity to heroin challenges lasted for 6 and 18 weeks, respectively. 

Interestingly, there has been substantial preclinical support for the fact that a single exposure to a drug of abuse could lead to long-lasting cellular neuroadaptations [37] as well as to long-lasting vulnerability to drug seeking behavior [38]. However, these “acute” addiction models do not completely mimic addiction in humans, which generally go through several relapses and remissions episodes [39]. Our results highlight the fact that numerous rather than a single episode of drug dependence augments the intensity of the long-lasting behavioral modifications [21]. The 1Dep group was subjected to heroin dependence and then to saline injections. It cannot be fully discarded that 1Dep animals treated with saline first and then heroin injections could have behaved such as 2Dep rats. However, the model of heroin dependence presented here is of importance for chronic treatment and presents an ideal model for longitudinal studies of the behavioral and neurobiological components associated with extended abstinence in rats. 

Previous studies have shown that chronic opiate administration with continued drug access leads to increased wakefulness during the sleep phase of the sleep/wake cycle in rats [13,40] as well as changes in circadian food intake [40]. Interestingly, the extended drug access also correlates with an escalation in drug intake [40]. Thus, it has been suggested that a dysregulation of circadian behaviors could contribute to the transition to opiate addiction. In our study, we show for the first time that protracted abstinence to opiate may also induce a long-lasting dysregulation of the circadian activity in rats. Sleep disruption and diurnal variation of the craving state profoundly affect protracted abstinent users [8,41]. Several neurobiological substrates have been suggested to contribute to circadian alterations after drug cessation. Recently, light has been shed on orexin, which is important in arousal, sleep as well as drug addiction [42,43]. In addition, this neuropeptide shows molecular adaptations to morphine and morphine withdrawal, and orexin knockout mice develop attenuated opiate dependence [43]. Thus, a possible hypothesis could be that persistent alterations of the circadian activity in our model of protracted heroin abstinence might be a consequence of long-lasting dysfunction of the orexin system.

Finally, the last experiment showed that the disruption of the diurnal activity in the 2Dep animals was greater at 16 and 20 weeks, suggesting an incubation-like phenomenon in the intensity of activity alterations. The term incubation was first used in both addicts and rat models of addiction to describe the observations that time-dependent increases in drug-seeking occurred after withdrawal from the drug [44,45]. The present study shows a rat model of heroin exposure that can be used to trigger incubation of circadian alterations mimicking such observations seeing in abstinent heroin users [8].

## 4. Materials and Methods

### 4.1. Animals

Adult male Sprague-Dawley rats (Charles River, Lyon, France) were collectively housed (3/cage) in a thermoregulated room (22 °C) with a 12:12 h light-dark cycle (light from 8 a.m. to 8 p.m.). Food and water were available ad libitum. These conditions were maintained constant throughout the experiments. All manipulations and observations were made during the light phase of the circadian cycle as follows: (i) for the induction of opiate dependence, saline/drug repeated injections were made during the light phase of the circadian cycle; and (ii) in the study of saline/heroin psychostimulant effects (experiment 1), saline/heroin challenges and locomotor activity monitoring were also performed during the light phase of the circadian cycle. However, when rats were monitored for spontaneous withdrawal (experiment 2) and circadian activity (experiment 3), the experimenter did not touch the animals. The experiments were carried out in accordance with the European Communities Council Directives (86/609/EEC, 24 November 1986) and the French Directives for the Use of Laboratory Animals (Decret 87–848, 19 October 1987).

### 4.2. Opiate Dependence Induction

Opiate dependence was induced either by (i) subcutaneous implantation of morphine pellets (2 × 75 mg basis), (ii) intraperitoneal (i.p.) injections of morphine (5 to 90 mg/kg/twice a day for 10 days) or (iii) i.p. injections of heroin (1 to 18 mg/kg/twice a day for 10 days). Injections were delivered at 8 a.m. and 6 p.m. Control animals were either implanted with placebo pellets (2 placebo pellets) or injected i.p. with saline (1 mL/kg/twice a day). The induction of opiate dependence took place in the home cages.

#### 4.2.1. Morphine Pellets (MP)

Two slow-release morphine pellets (75 mg of morphine base each, NIDA, USA) were implanted subcutaneously (lower back) under a rapid deep anesthesia (isoflurane/air; induction 4% v/v for 10 s followed by 1.5% v/v for 30 s). Under these conditions, we have shown that full dependence to morphine is achieved 24 h after implantation of the morphine pellets and remains constant for 15 days [46].

#### 4.2.2. Morphine Injections (MI)

Intraperitoneal injections of morphine sulfate (Coopération Pharmaceutique Française, France) were administered twice a day for 10 days according to the following schedule: 5 mg/kg the first day, 10 mg/kg the second day, and subsequent increase of 10 mg/kg/day to reach a dose of 90 mg/kg per injection on the 10th day. A last injection (90 mg/kg) was given on the morning of the 11th day. 

#### 4.2.3. Heroin Injections (HI)

Intraperitoneal injections of heroin sulfate (Coopération Pharmaceutique Française, France) were administered twice a day for 10 days according to the following schedule of escalating doses: 1 mg/kg the first day, 2 mg/kg the second day and subsequent increases of 2 mg/kg/day to reach a dose of 18 mg/kg per injection on the 10th day. A last injection (18 mg/kg) was given on the morning of the 11th day.

### 4.3. Activity Recording

Locomotor activity was measured in activity cages (35 × 25 × 25 cm) with wire mesh floors and 10 mm Plexiglas sidewalls (Imetronic, Pessac, France). Two infrared photoelectric cells were placed 14 cm apart and 3 cm above the floor. The activity cages were kept in a dimly lit room with a continuous white noise. Each experiment started with a habituation period, in which rats were placed into the activity chamber without any injection. 

### 4.4. Experimental Design

#### 4.4.1. Experiment 1: Long Lasting Sensitivity to Heroin Challenge Depends on the Previous History of Opiate Dependence

Animals were assigned to 4 groups according to the pharmacological approaches used to induce opiate dependence: placebo (control, *n* = 8), morphine pellets (MP, *n* = 8), morphine injections (MI, *n* = 8) and heroin injections (HI, *n* = 8). Abstinence was then induced by the pellets’ removal or cessation of the injections. Further, rats were tested for the locomotor response to heroin (0.25 mg/kg, s.c.) at several time points: 3, 6, 9, 12, 15 and 18 weeks of abstinence. For each challenge, rats were injected with saline (1 mL/kg, s.c.), monitored for 60 min in the activity cages; and the day after, injected with heroin and monitored for 180 min. All manipulations and observations were made during the light phase of the circadian cycle.

#### 4.4.2. Experiment 2: Repeated Episodes of Heroin Dependence: Consequences on the Circadian Activity upon Abrupt Cessation

Animals were allocated to 3 groups according to their heroin dependence history: placebo rats (control, *n* = 5), single episode of heroin dependence (1Dep, *n* = 7) and two episodes of heroin dependence (2Dep, *n* = 6). Dependence was induced by administration of escalating heroin doses in their home cages (See dependence induction paragraph: heroin injections), the 1Dep group received the treatment once, while the 2Dep group received two episodes of dependence, which were separated by a 2 weeks-interval. The control group received saline injections following the 2Dep group schedule. Subsequent to the last heroin injection schedule, opiate intoxication was abruptly terminated by ceasing the injections, and locomotor activity was monitored for 21 consecutive days. For this experiment, rats were permanently housed in the locomotor activity chambers during the spontaneous withdrawal, which allowed continuous recording. Temperature, dark-light cycle, and food and water availability were identical to those in the animal colony. Fluctuations of the circadian activity provide a well-established method for the characterization of spontaneous opiate withdrawal with rats’ biphasic activity being dramatically disrupted for some days [12].

#### 4.4.3. Experiment 3: Effects of Intermittent Exposures to Heroin: A Time-Course Analysis of the Circadian Activity throughout Extended Abstinence

Animals were allocated to 3 groups according to their heroin dependence history: placebo rats (control, *n* = 5), single episode of heroin dependence (1Dep, *n* = 7) and two episodes of heroin dependence (2Dep, *n* = 6). Dependence was induced by administration of escalating heroin doses once (1Dep) or twice (2Dep) as described above. The control group received saline injections following the 2Dep group schedule. Then, opiate withdrawal was induced by ceasing the injections. Subsequently, rats were repeatedly tested at 4, 8, 11, 12, 16 and 20 weeks of abstinence. On each test day, animals were placed in the locomotor activity chambers, and their general circadian activity was recorded for 24 h. Temperature, dark-light cycle, and food and water availability were identical to those in the animal colony.

### 4.5. Statistical Analyses

For all experiments, locomotor activity response was presented as the mean ± SEM photocell counts. The effects of opiate dependence method on saline- or heroin-induced psychomotor response were analyzed by two-way repeated measures analyses of variance (ANOVA), with Dependence (placebo, morphine pellets, morphine or heroin injections) as a between-subjects factor and Time as a within-subjects repeated-measures factor. The effects of repeated heroin dependence cycles on rats’ circadian activity were analyzed by a three-way repeated-measures ANOVA, with Circadian (daytime or nighttime cycle) as a within-subjects repeated-measures factor, Time as a within-subjects repeated-measures factor and Group (0, 1 or 2 heroin dependence episodes) as a between-subjects factor. All post hoc comparisons were made using Fisher’s PLSD tests. In all cases, differences with a *p* < 0.05 were considered significant.

## 5. Conclusion

In summary, while there are an increasing number of studies dedicated to the understanding of protracted abstinence, it remains unclear why it is difficult for individuals who have been previously dependent to maintain long-term abstinence [47]. Thus, the animal model of heroin dependence we have developed here provides an excellent research tool to study the underlying neurobiology of vulnerability to drug seeking behavior.
